# Transition Metal-Based 2D Layered Double Hydroxide Nanosheets: Design Strategies and Applications in Oxygen Evolution Reaction

**DOI:** 10.3390/nano11061388

**Published:** 2021-05-25

**Authors:** Birhanu Bayissa Gicha, Lemma Teshome Tufa, Sohyun Kang, Mahendra Goddati, Eneyew Tilahun Bekele, Jaebeom Lee

**Affiliations:** 1 Department of Chemistry, Chungnam National University, Daejeon 34134, Korea; ibs430@hotmail.com (B.B.G.); sohyunkang@naver.com (S.K.); 2 Department of Applied Chemistry, Adama Science and Technology University, P.O. Box 1888, Adama 1888, Ethiopia; lemmat2003@yahoo.com (L.T.T.); Eneyewtilahun77@gmail.com (E.T.B.); 3 Department of Chemical Engineering and Applied Chemistry, Chungnam National University, Daejeon 34134, Korea; gmahi066@gmail.com

**Keywords:** material design, transition metal layered double hydroxides, 2D nanosheets, oxygen evolution reaction

## Abstract

Water splitting driven by renewable energy sources is considered a sustainable way of hydrogen production, an ideal fuel to overcome the energy issue and its environmental challenges. The rational design of electrocatalysts serves as a critical point to achieve efficient water splitting. Layered double hydroxides (LDHs) with two-dimensionally (2D) layered structures hold great potential in electrocatalysis owing to their ease of preparation, structural flexibility, and tenability. However, their application in catalysis is limited due to their low activity attributed to structural stacking with irrational electronic structures, and their sluggish mass transfers. To overcome this challenge, attempts have been made toward adjusting the morphological and electronic structure using appropriate design strategies. This review highlights the current progress made on design strategies of transition metal-based LDHs (TM-LDHs) and their application as novel catalysts for oxygen evolution reactions (OERs) in alkaline conditions. We describe various strategies employed to regulate the electronic structure and composition of TM-LDHs and we discuss their influence on OER performance. Finally, significant challenges and potential research directions are put forward to promote the possible future development of these novel TM-LDHs catalysts.

## 1. Introduction

**Figure 1:**
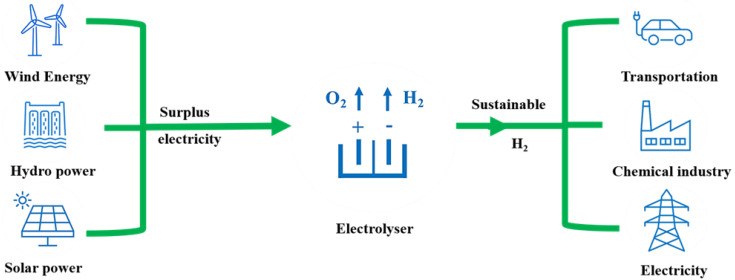
Overview of a sustainable hydrogen production from water electrolysis and its application in different sectors.

An increase in global demand for energy coupled with the rising environmental concerns related to fossil fuel use has motivated the world to search for alternative energy sources that are efficient, affordable, and environment friendly [1,2,3,4]. In this context, hydrogen, as an excellent energy carrier and abundant source with the highest energy per mass of any fuel, is considered an alternative low-carbon and sustainable energy source to replace fossil fuels [5,6,7]. Current hydrogen production is mainly sourced from fossil fuels involving CO_2_ production, which is not sustainable [8,9,10]. To address issues related to energy security, environmental pollution, and sustainability, searching for viable alternatives for hydrogen production that excludes emissions of any greenhouse gases is the key element. Among those, renewable energy-driven water splitting is one of the sustainable ways of hydrogen production, with a number of important applications (Figure 1), including the generation of clean electricity. Water splitting proceeds via two half-cell redox reactions; the reduction reaction is called the hydrogen evolution reaction (HER) [11,12] and the oxidation process is called the oxygen evolution reaction (OER) [13,14]. The OER is sluggish kinetically due to the process involving the formation of O–O double bonds, which requires the removal of four electrons with multiple intermediates, which, in turn, leads to a higher overpotential [15]. Therefore, a novel catalyst is desirable to reduce the higher overpotential and to facilitate the reaction kinetics of OERs.

Currently, outstanding electrocatalysts for HERs and OERs with prominent performances are based on Pt and RuO_2_/IrO_2_, respectively, which are expensive and scarce, limiting their extensive applications in scale-up progress [16,17]. Thus far, efforts have been dedicated to finding an economical and practical alternative to noble metal-based electrocatalysts. Among those alternative electrocatalysts reported for water electrocatalysis, first-row transition metal-based catalysts (TMs), such as Fe, Co, and Ni, have drawn considerable attention due to their improved activity [18,19,20,21]. Owing to their higher abundance, low cost, and environmental friendliness, TMs, and their oxides [22], hydroxides [23], chalcogenides [24], and phosphides [25], have shown potential applications as OER catalyst alternative to precious metals. Among various nonprecious metal-based OER electrocatalysts that have been studied so far, transition metal layered double hydroxides (TM-LDHs) with two-dimensional (2D) structures have invoked a lot of attention lately [26,27].

**Figure 2:**
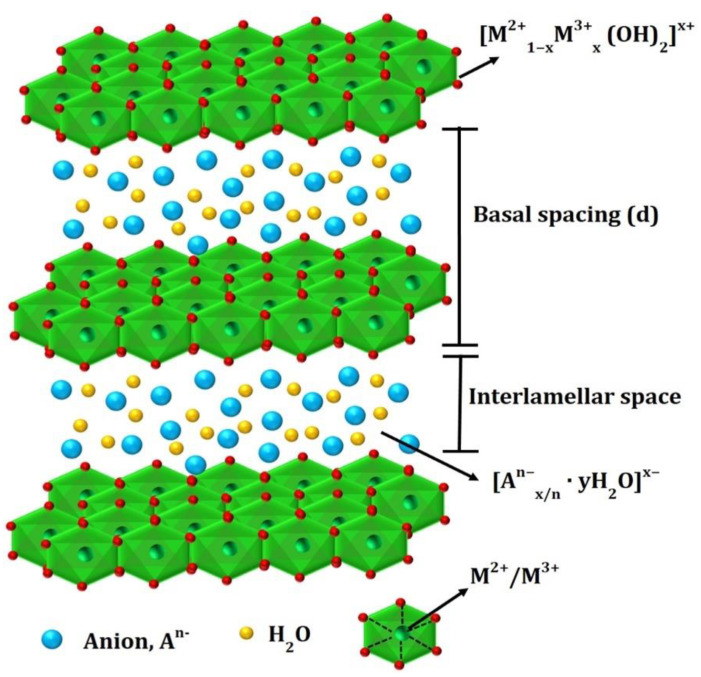
Typical structure of layered double hydroxide. Reproduced with permission from [40]. Elsevier, 2017.

LDHs are 2D anionic lamellar compounds with brucite-like host layers consisting of a positive charge and charge-balancing (exchangeable) interlayer anions, and they are represented as M^2+^_1−x_M^3+^_x_(OH)_2_(A^n−^)_x/n_·yH_2_O, where M^2+^ represents a divalent cation, M^3+^ is a trivalent metal cation, A^n−^ is a nonframework charge compensating an inorganic or organic anion, and the x value ranges between 0.2 and 0.4 (Figure 2) [28,29,30,31]. LDHs are characterized by positively charged [M^2+^_1−x_M_x_^3+^(OH)_2_]^q+^ layers where the interlayer region is occupied with the anions and water molecule [31]. The composition and electronic structure of TM-LDHs can be tuned through the accommodation of multiple metallic cations [32]. Furthermore, LDHs can be designed into a variety of structures, including single or few-layer nanosheets, exposing more active sites that, in turn, enhance their activity toward the OER [33]. Defects can be introduced through nanoscale porous engineering that enhances their OER performance by exposing the large number of electrochemically active sites [34,35,36]. Fewer active sites and poor electronic conductivity are the main challenges limiting the OER electrocatalytic performance of LDHs [37,38,39].

Numerous efforts have been dedicated to overcoming these limitations using different strategies such as doping of anions including sulfur [41], carbon [42], and nitrides [43], exfoliation into single or few-layer nanosheets [33], and engineering nanopores [35].

There have been many reports published on TM-LDHs in the past few years, mainly focusing on their synthesis and applications in energy conversion and storage. However, due to the immensity and rapid progress of the field, it is crucial to provide updated reviews for further improvement in the field. In this review, we present a snapshot of the recent advances in TM-LDHs for OERs mainly targeting newcomers to the field as opposed to earlier reviews, which are very exhaustive. Furthermore, this review focuses only on the first-row TMs (Ni, Co, and Fe) and their applications for OERs in alkaline conditions, making it concise and easy to understand. In addition, the overall progress from catalyst selection, design, to application is presented here, providing holistic information for further development in the field.

Herein, the recent progress and achievements on 2D TM-LDHs nanosheets and their applications in OER in alkaline conditions are briefly summarized. First, an overview of the electrocatalytic water splitting and mechanisms of OER in alkaline media is discussed. Then, fundamental parameters to evaluate electrocatalysts for efficient water electrocatalysis are discussed. After that, recent design strategies employed to improve the OER activities of 2D TM-LDH nanosheets, including morphological and microstructure engineering, composition tuning and electronic structure optimization, and hybridizing with conductive substrates to improve conductivity, are briefly reviewed. This work will enable readers to become familiar with the past and present advancements of this field while highlighting future prospects.

## 2. Electrocatalytic Water Splitting

### 2.1. Mechanism of OER in Alkaline Media

Electrocatalytic water splitting is an endothermic process and requires thermodynamic work to be done, requiring a potential equivalent to 1.23 V, theoretically [44]. Generally, water splitting proceeds via two half-cell reactions, in which hydrogen generates at the cathode and oxygen generates at the anode.

2H_2_O + Energy → 2H_2_ + O_2_ (1)

Practically, a higher voltage is required, and the process is more complicated. Generally, the two half-cell reactions in alkaline conditions can be expressed as follows:

2OH^−^ → 1/2O_2_ + H_2_O + 2e^−^ (Eanode = 1.23 V vs. RHE) (2)

2H_2_O + 2e^−^ → 2H_2_ + 2OH^−^ (Ecathode = 0.0 V vs. RHE) (3)

where Eanode and Ecathode are the equilibrium potentials for OER and HER in a reversible hydrogen electrode (RHE), respectively, at 25 °C and 1 atm.

**Figure 3:**
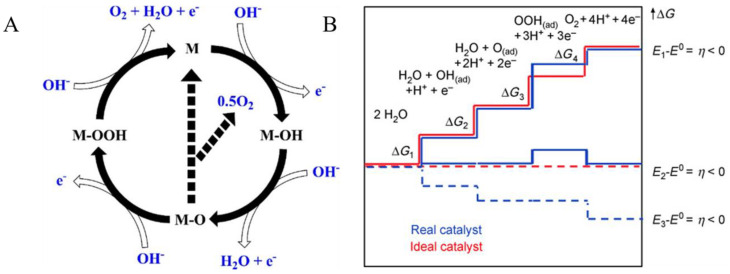
(**A**) Mechanism of OER, where M represents the active site. Reproduced with permission from [44]. American Chemical Society, 2018. (**B**) Plots of Gibbs free energy of reactive species and intermediates of the OER (horizontal lines) vs. the reaction coordinate. At three different electrode potentials, the blue and red lines represent the energetics of a real catalyst and an ideal catalyst, respectively. The energetics at the electrode potential where all thermochemical barriers disappear (“thermoc”) are indicated by dashed lines. Reproduced with permission from [45]. WILEY-VCH Verlag GmbH & Co. KGaA, Weinheim, 2018.

In water electrolysis, the OER is a limiting reaction requiring a higher overpotential than the equilibrium potential compared to the HER, which can proceed at a potential close to its equilibrium potential. Understanding the mechanisms of HER and OER is paramount in designing high-performing electrocatalysts that can deliver high current densities. In contrast to HER, OER involves four-electron transport process [44], as shown in Figure 3. Here, the intermediates involved are represented as O*, HO*, and HOO*, whereas the active site is represented as “M”. On the active site, OH^−^ is adsorbed to give M–OH. M–O is produced from M–OH through the removal of a coupled proton and electron. As can be seen from Figure 3A, two different pathways lead to O_2_ formation. The first pathway involves the reaction between M–O and OH^−^, leading to the formation of a M–OOH intermediate, followed by active site regeneration through the deprotonation of M–OOH, which leads to O_2_ formation. The second pathway involves a combination between the two M–O species, leading to the formation of O_2_ and M. Compared to the first pathway, the second pathway is considered to have a large activation barrier.

The Gibbs free energy changes (∆G) obtained during the process of chemical reaction also provides additional information regarding the intrinsic activities and kinetics of a given catalyst (Figure 3B). The Gibbs energy for an ideal catalyst and the chemosorption energies are equal for each step (∆G_1_ = ∆G_2_ = ∆G_3_ = ∆G_4_), whereas, in the case of real catalysts, that is not true, where the values can be assumed as: (∆G_3_ > ∆G_1_ = ∆G_2_ > ∆G_4_). For both ideal and real catalysts, the OER cannot proceed at the electrode potential (E1), because of the higher overpotential required at this step. The value of ∆G_3_ remains positive for real catalysts, while other intermediates changes to positive or diminish, indicating that the catalyst surface interaction of OOH(ad) is weaker. Therefore, the design of OER catalysts with improved performance should consider M-O binding optimization within the intermediates [46].

**Figure 4:**
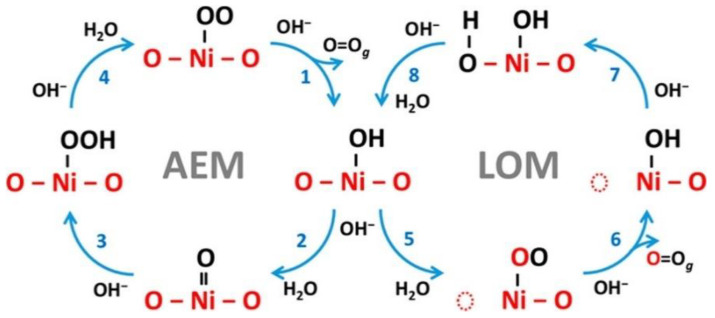
Demonstration of competition between the adsorbate evolution (AEM) and the lattice-oxygen mechanism (LOM). Reproduced with permission from [49]. American Chemical Society, 2018.

The most common OER mechanisms based on thermodynamics can proceed through the following two mechanisms: lattice oxygen-mediated mechanism (LOM) and adsorbate evolution mechanism (AEM) (Figure 4) [47]. In the AEM, the process involves a four-electron transfer where all the metallic active sites facilitate the reaction of oxygen intermediate species, resulting in a decrease of overpotential. The fundamental steps for AEM can be described as follows in alkaline conditions [48]:

4OH^−^ → OH* + 3OH^−^ + e^−^ (4)

OH* + 3OH^−^ + e^−^ → O* + 2OH^+^ + H_2_O + 2e^−^ (5)

O* +2OH^−^ + H_2_O + 2e^−^ → OOH* + OH^+^ + 2H_2_O+ e^−^ (6)

OOH* + OH^−^ + 2H_2_O + 3e^−^ → O_2_(g) + 2H_2_O + H+ + 4e^−^ (7)

In the LOM, scaling limitations occurring in the AEM improves and, basically, it involves the oxidation of lattice oxygen. The acidic-based LOM can be described as follows [48]:

H_2_O + * → OH* + H^+^ + e^−^ (8)

OH* → O* + H^+^ + e^−^ (9)

O* + O_L_ → O_2_ + V_o_ (10)

V_o_ + H_2_O → OH* + H^+^ + e^−^ (11)

H* → * + H^+^ + e^−^ (12)

where * is an active site, O_L_ is lattice oxygen, and V_o_ is a surface oxygen vacancy.

### 2.2. Fundamental Parameters to Evaluate Electrocatalysts

Selecting and evaluating electrocatalysts for large-scale water electrolysis is fundamental in the development process of a sustainable energy future. To evaluate the performance of a given catalyst, the following basic criteria are widely recognized.

#### 2.2.1. Overpotential (η)

Electrochemical reactions cannot proceed at 1.23 V vs. RHE, known as the thermodynamic potential, without considering the kinetic hindrances encountered in a real system [50]. Therefore, an externally applied potential is required to overcome reaction barriers, which is called the overpotential. Based on the Nernst equation (Equation (13)), the applied potential for an electrocatalytic reaction can be calculated as [39]:

(13) $$E=E^{0}+\frac{RT}{nF}ln\frac{\left[Ox\right]}{\left[Red\right]}$$

where *E* is the potential, *E*^0^ is the standard potential, *R* is the ideal gas constant, *T* is the absolute temperature (K), *n* is the moles of electrons, *F* is the Faraday constant, [*Red*] is the reduced molecules (moles), and [*Ox*] is the oxidized molecules (moles).

Generally, the overpotential can be estimated as follows:

(14) $$\eta =E-E_{eq}$$

where *ƞ* is the overpotential, *E* is the applied potential, and *E_eq_* is the equilibrium potential.

#### 2.2.2. Tafel Slope (b)

The Tafel equation is one of the fundamental parameters in electrolysis, formulating a quantitative relationship between the current and applied potential [51]. It depicts how the current responds sensitively to an overpotential. It is derived from the polarization curve as a plot of the logarithm of current density (*log*(*j*)) versus *η*. In the Tafel plot, the linear correlation between the Tafel slope (*b*) and the exchange current density (jo) is expressed. The Tafel slope can be estimated using the following equation, where *b* represents the Tafel slope:

(15) η=b log(j)+a

A low Tafel slope value is an indication of fast reaction kinetics, characteristics of good OER electrocatalysts [52]. The Tafel slope enables us to elucidate the reaction mechanism of electrocatalysts, providing information about the rate-determining step [53].

#### 2.2.3. Turnover Frequency (TOF)

It is important to understand the specific activity of a given catalyst to gain insight into its activity differences based on different mass loadings. *TOF* can be described as the rate at which electrons are delivered per surface metal atom per second or it is the rate at which molecules evolve per active site per unit time [54]. As a measure of catalyst efficiency, *TOF* can be described as the number of molecules converted per active site per unit time [44]. *TOF* can be calculated as follows:

(16) $$TOF=\frac{J\times A}{4\times F\times n}$$

where *J* (mA cm^−2^) is the measured current density at a given overpotential, *A* is the surface area of the catalyst, *F* is the Faraday constant, and *n* is the number of moles of the active materials. For economical and practical applications, catalysts with *TOF* values are highly required because of their short reaction time.

#### 2.2.4. Electrochemical Surface Area (ECSA)

As water electrolysis is a surface reaction taking place at active sites, *ECSA* is a key parameter to evaluate the activity of a given catalyst. As the area is more exposed, so are the active sites enhancing higher mass transfers, facilitating the rate of reaction. It will allow us to determine quantitatively the reacting interface of a given catalyst [55]. The most common method of calculating *ECSA* is based on the electric double layer capacitance (*C_dl_*). The *C_dl_* can be estimated by measuring cyclic voltammograms (CVs) in a non-Faradaic potential region at different scan rates. Once *C_dl_* is estimated, the *ECSA* can be estimated as:

(17) $$ECSA=\frac{C_{dl}}{C_{s}}$$

where *C_s_* is the specific capacitance.

#### 2.2.5. Stability

For commercial applications, the long-term stability of a given catalyst is top priority. Generally, the OER catalyst stability can be evaluated by measuring the change in activity or change in physicochemical properties of the catalyst. The activity changes can be measured electrochemically using chronoamperometry/chronopotentiometry and cyclic voltammetry. The cyclic voltammetry reported usually ranges from 250 to 1000 cycles for OER catalyst stability tests. We can also check stabilities using chronoamperometry (fixed potential) or chronopotentiometry (fixed current) running for a given period of time. Given that, a stable current density of 10 mA cm^−2^ or a constant overpotential at 10 mA cm^−2^ over a period of the stability test is an indicator of the material’s durability. Another technique of evaluating durability includes the analysis of spent electrolytes using inductively coupled plasma mass spectrometry (IPC-MS).

## 3. Design Strategies of TM-Based LDHs for Improved OER Catalysis

The TM-LDHs are potential candidates for electrocatalysis because of their unique features, including their flexibility to incorporate metals of different valence states. However, they commonly suffer from poor catalytic performance, mainly attributed to their low conductivity and insufficient exposure of active sites with poor intrinsic activities. To overcome those challenges, several design strategies have been developed and employed.

### 3.1. Structural and Morphological Engineering

It is well-known that the performance of OER electrocatalysts is highly dependent on the degree of exposure of active sites, which, in turn, is directly affected by the structural and morphological properties of the catalyst material [56,57,58]. Several strategies have been employed to optimize the morphology of LDHs such as exfoliation [59,60,61,62], creation of defects and pores [63,64], and alteration of the assembly of the surface structures [59,65].

#### 3.1.1. Exfoliation

Exfoliation is a technique employed to delaminate atomically stacked layers of LDHs into a single nanosheet layer with an ultrahigh specific area and to expose more active sites that are available at the surface-active catalytic materials [66]. Accordingly, the exfoliated materials exhibit unique electrical, chemical, and optical properties compared to their counter bulk materials. Various exfoliation approaches such as liquid- and dry-based have been used in the past years.

In the liquid-type exfoliation, the solvent molecule interacts with the layers of bulk TM-LDHs, enlarges the basal spacing, and decreases the interaction between the metal layer and anions, allowing the formation of thin nanosheets. The first exfoliation strategy was employed by Song and Hu using liquid exfoliation method to enhance the activity of NiFe-, CoCo-, and NiCo-LDHs for OER catalysis [59]. With this strategy, they were able to produce single-layer nanosheets with a higher OER performance without altering the composition and structure of the bulk LDHs. Compared to bulk LDHs, the exfoliated nanosheet performances have improved by 2.6-, 3.4-, and 4.5-fold for CoCo, NiCo, and NiFe-LDHs, respectively, for OER catalysis. Furthermore, Zhou et al. also adopted the liquid-based exfoliation technique to exfoliate CoFe LDHs to a single layer with multiple vacancies [67]. They used HNO_3_ to destroy the strong attraction between host layers and interlayer anions, leading to the formation of a single layer. The acid-etched CoFe LDHs have demonstrated a higher OER performance compared to bulk CoFe LDHs, owing to its creation of abundant vacancies of Co, Fe, and O with multiple defects, offering more active sites.

**Figure 5:**
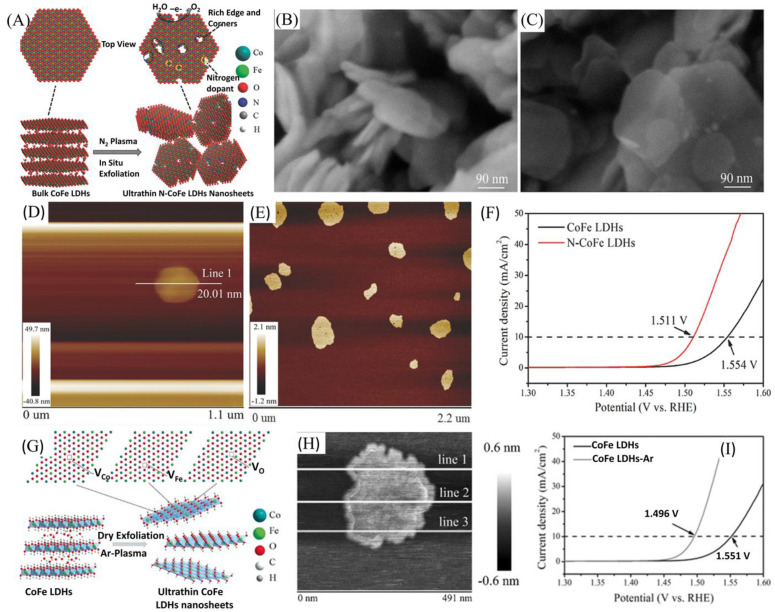
(**A**) Illustration of the exfoliation process of bulk CoFe LDHs into ultrathin CoFe LDH nanosheets by N plasma. (**B**) Scanning electron microscope (SEM) image of bulk CoFe LDHs. (**C**) SEM image of ultrathin N-CoFe LDH nanosheets. (**D**) Atomic force microscopy (AFM) image of bulk CoFe LDHs. (**E**) AFM image of ultrathin N-CoFe LDH nanosheets. (**F**) Linear sweep voltammetry (LSV) polarization curves of bulk CoFe LDHs and ultrathin N-CoFe LDHs nanosheets at a scan rate of 5 mV s^−1^. Reproduced with permission from [71]. WILEY-VCH Verlag GmbH & Co. KGaA, Weinheim, 2017. (**G**) Schematic illustration of exfoliation of CoFe LDH nanosheets by Ar-plasma. (**H**) AFM image of ultrathin CoFe LDHs-Ar nanosheets. (**I**) LSV curves for the OER at a scan rate of 5 mV s^−1^. Reproduced with permission from [69]. WILEY-VCH Verlag GmbH & Co. KGaA, Weinheim, 2017.

Despite its advantages, liquid-based exfoliation may suffer from the restacking of LDHs after the removal of exfoliating liquid, and there is also a probability of the adsorption of solvent molecules to LDHs, reducing its activity by blocking surface active sites [68,69]. Owing to its environmental friendliness, short treatment time, and ease of introduction of active species, plasma exfoliation has risen as an alternative method to exfoliate LDH materials [69,70]. Wang et al. employed N plasma to exfoliate bulk CoFe LDHs into edge-rich ultrathin LDH nanosheets (Figure 5A–F) [71]. The exfoliation process led to the formation of multiple pores, enhancing the surface area, and creating rich edges with a thickness of only 1.6 nm (Figure 5E). Furthermore, the doping of N led to electron rearrangement in the reactive site, which, on the other hand, facilitates the adsorption of OER intermediates. Owing to its electronic and structural changes, the N-CoFe LDH nanosheet has demonstrated the lowest overpotential of 281 mV compared to bulk CoFe LDH (324 mV) to generate a current density of 10 mA cm^−2^ (Figure 5F). In addition to a change in the bulk layer to single layer in LDHs, the creation of vacancies could also lead to improved OER activities through enhancing the exposure of active surface atoms [72]. In this regard, Wang et al. successfully exfoliated and introduced multiple vacancies into CoFe LDHs using Ar-plasma etching technique (Figure 5G). Compared to bulk CoFe LDH, the ultrathin 2D CoFe nanosheets exhibited improved OER activity. With the application of Ar-plasma, they were able to break the interlayer bonds in the bulk LDHs, resulting in the formation of a single layer of 0.68 nm in thickness (Figure 5H). With this process, multiple vacancies were created that would, in turn, tune the electronic structure and property of the material, hence enhancing its OER activity. The CoFe LDHs-Ar nanosheet exhibited a lower overpotential of 266 mV at 10 mA cm^−2^ compared to bulk CoFe LDH that required 321 mV at the same current density (Figure 5I). Furthermore, CoFe LDHs-Ar nanosheets showed a minimum Tafel slope of 37.85 mV dec^−1^, an indication of a faster kinetic process in contrast to bulk CoFe LDHs that showed a higher Tafel slope of 57.05 mV dec^−1^. Added to these, Liu et al. also demonstrated an enhancement of the catalytic activity of NiCo-LDHs by exfoliating using Ar-plasma [62]. The as-obtained NiCo-LDHs/Ar nanosheet was only 1.1 nm in thickness with richer defects compared to bulk NiCo-LDHs. As a result, the NiCo-LDHs/Ar nanosheet has demonstrated a higher activity with an overpotential as low as 299 mV at a current density of 10 mA cm^−2^ and fast reaction kinetics with a low Tafel slope of 45 mV dec^−1^ compared to pristine NiCo-LDH. A comparison of the OER activities of exfoliated TM-LDHs is given in Table 1.

There are also other exfoliation strategies, including solid-state exfoliation, Ostwald ripening exfoliation, and supercritical ethanol exfoliation that can also be employed to optimize the activity of TM-LDHs [75]. For instance, Li et al. employed the solid-phase exfoliation strategy to obtain thin layers of nanosheets from NiFe LDHs and graphene oxide (GO) [76]. The exfoliated heterostructure nano-hybrids demonstrated a higher activity with a Tafel slope of 49 mV dec^−1^ and a lower overpotential of 273 mV at a current density of 30 mA cm^−2^, benefiting from the enhanced charge transfer and exposure of active sites. Ostwald ripening, a process of the formation of large particles from the dissolution of smaller particles to reach equilibrium, can also be employed to exfoliate bulk LDHs to thin nanosheets. Chen et al. used the Ostwald ripening-driven exfoliation strategy to exfoliate NiFe LDHs into ultrathin and vertically aligned nanosheets, which resulted in an enhanced activity as a result of the increase in surface area and the exposure of active sites and edges. This technique is simple, and the process does not require any exfoliating reagent or surfactant, and it can be applied to other 2D LDHs for novel applications [60]. The exfoliated ultrathin NiFe nanosheets enhanced the OER performance with a low overpotential of 292 mV to reach a current density of 10 mA cm^−2^ and a long-term stability of more than 60 h. Ma et al. developed Ni^2+^Mn^3+^ LDHs through organic anion exchange liquid-exfoliation method [77]. The exfoliated nanoplate exhibited a regular lamellar stacking feature with an interlayer spacing of ∼0.80 nm and a thickness of approximately 10 nm, as visualized from high-resolution transmission electron microscopy (HRTEM). The as-prepared NiMn LDH nanosheets exhibited an overpotential of 0.36 V for oxygen evolution catalysis in 1 M KOH.

#### 3.1.2. Defect Engineering

**Figure 6:**
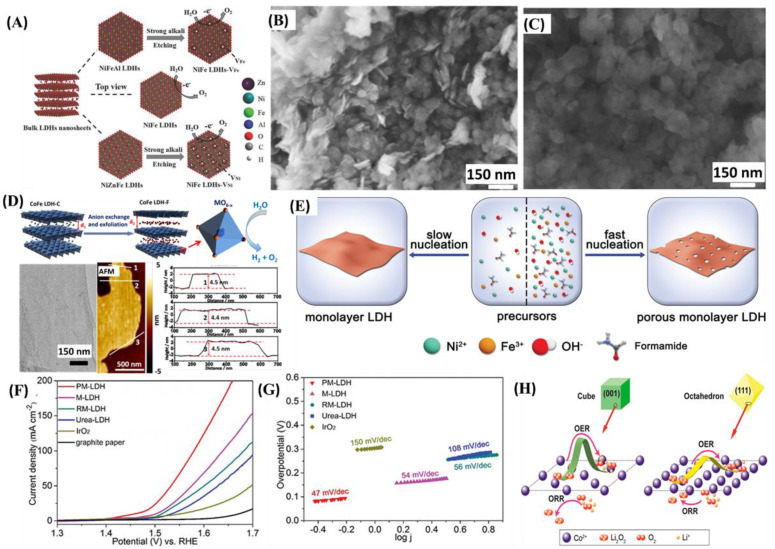
(**A**) The synthesis of NiFe LDHs-V_Fe_ and NiFe LDHs-V_Ni_ by strong alkali etching LDHs. (**B**) SEM image of NiFe LDH-V_Fe_. (**C**) SEM image of NiFe LDH-V_Ni_. Reproduced with permission from [86]. WILEY-VCH Verlag GmbH & Co. KGaA, Weinheim, 2018. (**D**) Schematic illustration of exfoliation of CoFe LDH, and transmission electron microscopy (TEM) and AFM images. Reproduced with permission from [89]. American Chemical Society, 2016. (**E**) Schematic illustration of synthesis of porous monolayer LDH and monolayer LDH. (**F**) LSV curves at a scan rate of 5 mV s^−1^. (**G**) Corresponding Tafel slopes. Reproduced with permission from [90]. WILEY-VCH Verlag GmbH & Co. KGaA, Weinheim, 2019. (**H**) Schematic illustration of the facet-dependent electrocatalytic mechanism of ORR and OER on Co_3_O_4_ (001) and Co_3_O_4_ (111), respectively. Reproduced with permission from [94]. American Chemical Society, 2015.

The engineering of LDH materials with rich edge sites is critical toward developing and designing improved catalytic materials. Introducing more active sites that improve the catalytic activity of materials is the center of focus in the fields of catalysis [78,79]. One of the methods employed to increase the number of active sites is through structural defect engineering of the catalyst material via generating massive electrochemically active grain boundaries in the TM oxides [80,81]. Different strategies have been used to create defects in LDHs, such as doping [82], morphological tuning [83], etching [84], and controlled growth [85]. Wang et al. were able to synthesize Ni^2+^ or Fe^3+^ defect-rich NiFe LDHs nanosheets [86]. The process involved the synthesis of NiFeAl-LDHs and NiZnFe-LDHs using the conventional hydrothermal technique, followed by etching using a strong alkali, resulting in the creation of defects (Figure 6A–C). The creation of defects in NiFe LDH nanosheets subsequently altered the surface electronic properties, improving their OER activities. As a result of creation of defects, NiFe LDHs-VFe and NiFe LDHs-VNi have demonstrated lower overpotentials of 245 and 266 mV, respectively, in contrast to bulk NiFe LDHs that exhibited an overpotential of 299 mV to generate a current density of 10 mA cm^−2^. In addition to cationic defects, the introduction of anionic defects, including oxygen vacancies, can enhance the activity of a given material through facilitating the adsorption of OH^−^ intermediates [87,88]. Liu et al. were able to improve both the HER and OER catalytic activities of bulk CoFe LDH through delamination and exfoliation [89]. They first synthesized bulk CoFe LDHs using hydrothermal technique, and the material was treated in a mixture of dimethylformamide (DMF)/ethanol to obtain ultrathin CoFe-LDHs rich in defects (Figure 6D). The process led to the formation of a 0.8 nm ultrathin nanosheet (Figure 6D), demonstrating activity enhancement requiring a lower overpotential of 300 mV at a current density of 10 mA cm^−2^. The enhanced activity is attributed to the creation of defects and improved conductivity of the electrode material. Zhang et al. showed that the defect-rich ultrathin Co(OH)_2_ nanoarray exhibited an OER activity 3–4 times higher than that of commercial RuO_2_ [90]. A defect-rich, porous NiFe-LDH monolayer, only 0.8 nm in thickness, was synthesized, as demonstrated in Figure 6E. Owing to its rich defects and high porosity, NiFe-LDH showed a higher catalytic activity (Figure 6F,G). To manipulate the coordinately unsaturated metal sites in NiFe LDHs, Wu et al. employed a defect engineering strategy [91]. They used fluoride adsorbate to cover metal sites upon preparation and later remove electrochemically to control unsaturated metal sites on NiFe LDHs, which was used as an OER electrocatalyst. The optimized fluoride-pre-covered NiFe LDH exhibited a higher catalytic activity, requiring an overpotential of only 243 mV at a current density of 10 mA cm^−2^.

#### 3.1.3. Facet Engineering

Facet engineering of nanostructured materials is one of the strategies employed to optimize catalytic activities through enhancing the exposure of more active sites [92]. The rational design and development of a new catalyst material with active facets, favorable atomic structure, and coordination is the most promising approach in exposing highly reactive sites toward accelerating surface kinetics [93]. Reports have indicated that variations in the exposed facets within the same material can lead to different catalytic performances [94]. Gao et al. investigated the facet-dependent performances of well-defined Co_3_O_4_ cubes through a combination of both experimental and theoretical studies [94]. They determined experimentally that Co_3_O_4_ octahedra with exposed (111) planes demonstrated a higher performance compared to Co_3_O_4_ cubes with exposed (001) planes, because of the more exposed active sites and richer Co^2+^. Furthermore, they employed density functional theory (DFT)-based calculations to understand the interaction mechanisms of Co_3_O_4_ planes with Li_2_O_2_ in the OER and oxygen reduction reaction (ORR) processes (Figure 6H). The theoretical calculations revealed that Co_3_O_4_ (111) has a lower activation barrier of O_2_ desorption in the OER.

In their recent work, Zhang et al. reported how the catalytic activity of LDHs are affected by the exposure of facets [95]. The hydrothermally synthesized NiFe-LDH has facets of the (011) basal plane and (100 and 110) facets corresponding to edge facets. The electrochemical characterization results showed that there is a direct relationship between the increase in edge area ratio and the enhanced electrochemical activity. The overpotential decreased from 350 to 346 mV as the edge area ratio increased from 8.6% to 9.2% at a current density of 10 mA cm^−2^. Furthermore, the Tafel slope also showed a positive correlation with edge area. As the edge area ratio increased from 8.60% to 9.59%, the Tafel slope decreased from 73.5 to 57.4 mV dec^−1^.

#### 3.1.4. Interfacial Engineering

As a design strategy, interfacial engineering is evolving because of the interaction between different components can be employed to enhance the activity and stability of TM-LDHs [96]. Interfaces in the catalysis system are areas of concern as they influence the activity and stability by adjusting the surface adsorption of intermediates and the transportation of electrons [97]. Interfacial engineering can improve the performance of electrocatalysts through enhancing their activity, selectivity, and stability, which are fundamental features of a given catalyst. For instance, Anantharaj et al. synthesized Pt nanoparticles (NPs)-decorated NiFe LDHs for overall water electrocatalysis under alkaline conditions [98]. An interface between NiFe LDHs and Pt NPs was created by the reduction of Pt^4+^ in borohydride solution containing NiFe LDH nanosheets. The Pt NPs-decorated NiFe LDH nanosheet demonstrated a higher activity, indicating the potential contribution of the interface formed between the two layers towards OER enhancement. It was reported that the interaction between metal atoms and TM-LDHs is an ideal way to enhance activities, which creates favorable interfacial interactions [99,100]. Zhang et al. designed an Au-supported NiFe LDH as an OER catalyst under alkaline conditions [99]. The material demonstrated excellent OER activity, which is mainly attributed to the interfacial charge transfer between Au and NiFe LDH. DFT calculations also revealed that CO_3_^2−^ ions in the interlayer between Au-supported oxyhydroxides and LDHs could balance the charges of oxyhydroxide, leading to the enhanced adsorption of intermediates on the surfaces of Fe.

### 3.2. Composition Tuning and Electronic Structure Optimization

As a strategy to tune compositions and electronic structures of electrocatalysts, doping has been used widely owing to its effective role in enhancing active sites and reducing energy barriers [101]. Compared to undoped electrocatalysts, doped electrocatalysts have demonstrated better performances benefiting from heterostructures [102]. Recently, a catalytic activity improvement was observed on heteroatom-doped electrocatalysts obtained through the incorporation of metallic cations such as Fe [103], Co [104], and Ni [105] and anions such as S [106], N [107], P [108], and O [109]. Furthermore, heteroatom doping does not change the composition of host materials, retaining the desired intrinsic features [110]. The applausive achievements of doped materials paved the way for the advancement of nonnoble metal-based nanomaterials as highly efficient electrocatalysts [101].

**Figure 7:**
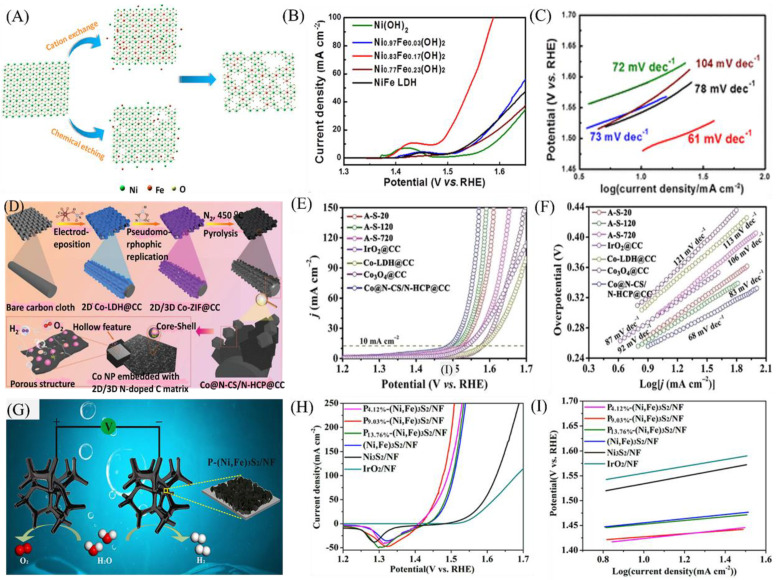
(**A**) Schematic illustration of the preparation of Fe-doped Ni (OH)_2_ nanosheets. (**B**) LSV curves of Ni_0.83_Fe_0.17_(OH)_2_ nanosheets. (**C**) Tafel slopes of Ni_0.83_Fe_0.17_(OH)_2_ nanosheets. Reproduced with permission from [114]. American Chemical Society, 2018. (**D**) Schematic illustration of the synthesis of Co@N-CS/N-HCP@CC composite. (**E**) LSV curves. (**F**) Corresponding Tafel plots. Reproduced with permission from [126]. WILEY-VCH Verlag GmbH & Co. KGaA, Weinheim, 2019. (**G**) Illustration of water splitting on the surface of P-(Ni, Fe)3S2/NF. (**H**) LSV curves. (**I**) Corresponding Tafel plots. Reproduced with permission from [128]. American Chemical Society, 2019.

Cationic doping can be regarded as an efficient way to tune the morphology and electronic structure of LDHs, where metal atoms with a higher valence state can act as dopants [111,112]. Recently, Zheng et al. proposed that the doping of Fe^3+^ into cobalt-based LDH nanosheets can tune the Co^2+^ occupancy and coordination [113]. It was believed that the Fe^3+^ dopant can regulate the coordination of Co^2+^ in CoO_4_ tetrahedra and CoO_6_ octahedra. The as-synthesized CoFe LDH nanosheets with an optimized ratio of 5:1 (Co/Fe) has demonstrated an overpotential of 285 mV at a current density of 10 mA cm^−2^ and a Tafel slope of 44.6 mV dec^−1^. Furthermore, Zhou et al. proposed the cation-exchange method to synthesize an active site-rich OER electrocatalyst based on Fe-doped Ni(OH)_2_ and NiFe LDHs (Figure 7A) [114]. The as-synthesized Fe-doped Ni_0.83_Fe_0.17_(OH)_2_ nanosheets with abundant defects and a porous structure demonstrated a lower overpotential (245 mV) at a current density of 10 mA cm^−2^ and enhanced reaction kinetics with a lower Tafel slope of 61 mV dec^−1^ in contrast to NiFe LDH nanosheets prepared by the conventional method (Figure 7B,C). The improved activity was from the potential contribution of the cation exchange method, resulting in enriched defects and actives sites. Liu et al. proposed cationic doping as a strategy to alter the surface chemical environment of an electrocatalyst for enhanced OER activity [111]. They prepared ultrathin Ni_3_FeAlx LDH nanosheets using hydrothermal technique with the introduction of Al that led to a change in the fraction of low-coordinated Fe and Ni atoms. The result shows that Ni_3_FeAl_0.9_ LDH demonstrated an improved activity with a measured overpotential of 304 mV at a current density of 20 mA cm^−2^ and a Tafel slope of 57 mV dec^−1^, an indicator of fast reaction kinetics. Furthermore, Thenuwara et al. also systematically incorporated Co into NiFe LDHs for enhanced OER catalysis using coprecipitation and/or intercalation [115]. They proposed that Co intercalation into the interlayer of NiFe LDHs can increase its activity. The result indicates that Co-modified NiFe LDHs showed an outstanding OER activity with a lower overpotential ranging between 290 and 322 mV at a 10 mA cm^−2^ current density depending on the amount of Co incorporated.

Besides cation doping, anion doping also has been implemented as a potential design strategy to increase the catalytic performance of TM-LDHs [106]. Anion dopants such as N, P, F, and S (Table 2) have the potential to enhance the activity of the host material via changing the electronic structure and conductivity, decreasing the adsorption/desorption energy during water electrolysis [116,117]. Among anion dopants, sulfide is praised for its potential in enhancing electronic conductivity and electrocatalytic activity, with minimal environmental effects [118,119,120]. Shit et al. fabricated a heterostructure sulfur-doped electrocatalyst for overall water splitting directly grown on Ni foam with an enhanced performance [121]. Benefiting from the heterostructure and porous 3D Ni foam support material, the as-obtained material demonstrated comparable catalytic performance with benchmarking catalysts. The incorporation of CoSx is believed to enhance the number of catalytically active sites, and the value was determined to be 1.993 × 10^18^ atoms cm^−2^, increasing by a factor of 2.92 times after CoSx incorporation. Furthermore, the Co-S-Ni moiety enhances the activity of the catalyst material by improving the charge transfer efficiency. The porous heterostructure CoSx/Ni_3_S_2_/NF has achieved a current density of 20 mA cm^−2^ at an overpotential of 280 mV for OER catalysis.

Heteroatom doping with those having different electronegativities can adjust the electron distribution of LDHs, leading to superior OER activity. In this regard, anion-doped metal−organic frameworks are emerging classes of materials with multiple catalytic benefits, including high surface area, porous structure, and tailorable features [125,126]. For instance, Chen et al. designed a heterostructure metal−organic framework as an efficient electrocatalyst for OER catalysis [126]. They were able to synthesize (zeolitic imidazolate framework, ZIF-67) 2D ZIF-67/CC nanosheets by using Co-LDH and a 3D ZIF-67 precursor (Figure 7D). The as-obtained 2D ZIF-67/3D ZIF-67 transformed into Co@N-CS/N-HCP@CC upon heating to a lower temperature. The material has demonstrated a higher catalytic activity benefiting from its minimum charge transfer resistance, fast reaction kinetics, and rich active sites. As can be seen from Figure 7E, Co@N-CS/N-HCP@CC showed a higher OER activity, requiring an overpotential of 248 mV to generate a current density of 10 mA cm^−2^. The lower recorded Tafel slope of 68 mV dec^−1^ is also an indication of faster reaction kinetics of the catalyst material (Figure 7F). The material has also shown improved charge transfer abilities with measured charge transfer resistances (Rct) as low as 16.7 Ω. Furthermore, DFT calculations also revealed that the synergistic effect between C and Co led to the improved adsorption of active sites toward intermediates compared to pristine C, resulting in enhanced catalytic activity.

Another potential anion dopant is P, where its incorporation into TM-LDHs improves the conductivity and intrinsic activities. Previous studies have demonstrated, through DFT calculations, that the higher electronegativity of P than S could influence the electronic structure and result in a shift in the d band center, which could possibly optimize ∆GH* [127]. For instance, Liu et al. designed P-doped 3D P-(Ni, Fe)_3_S_2_/NF through the incorporation of P and S simultaneously as an efficient electrocatalyst for water electrolysis (Figure 7G) [128]. In alkaline media, the as-obtained material has demonstrated good OER activity compared to the undoped material. An overpotential of 196 mV has been recorded at a current density of 10 mA cm^−2^ for P_9.03%_-(Ni, Fe)_3_S_2_/NF. The electrocatalyst also has a lower Tafel slope of 30 mV dec^−1^ and robust stability, mainly attributed to surface oxidation during electrolysis, characteristics of TM-based sulfides, nitrides, and phosphides (Figure 7H,I) [129]. 

To further confirm the role of P doping on the catalytic activity of the (Ni, Fe)_3_S_2_, DFT calculations were carried out. DFT calculations showed that the incorporation of P into (Ni, Fe)_3_S_2_ resulted in the reduction of ∆GH* values by −0.1 eV compared to the undoped material, indicating improved catalytic activity.

### 3.3. Hybridizing with Conductive Substrate

Different strategies such as structural and electronic property optimization have been applied to overcome the poor conductivity of LDHs. The low conductivity of the catalysts coupled with their difficulty for practical applications has necessitated the hybridization with conductive substrates such as nickel foam and carbon nanomaterials [112,130]. Producing catalysts by directly growing on conductive substrates enhances the long-term stability, providing mechanical strength, optimizes the electrochemically active surface area, and facilitates diffusion and the fast emission of products.

The use of commercial conductive support materials such as Ni foam has been the center of interest in heterogeneous catalysis owing to its flexible uses [131]. Wang et al. designed self-supported NiVIr-LDH and NiVRu-LDH on Ni foam as catalysts for water electrolysis [132]. The as-obtained material has shown excellent catalytic activity for the HER and OER, demonstrating excellent exchange current densities and TOF. The direct growth on the conductive substrate (Ni foam) improved the conductivity of the catalyst material. Li et al. designed partially crystalline NiFe LDH nanosheets on Ni foam as an efficient water splitting catalyst through a facile and sustainable approach [133]. Due to the 3D open architecture grown vertically on the porous NF surface, the NiFe-LDH/NF demonstrated an outstanding OER performance with a lower overpotential of 133 mV to generate a current density of 20 mA cm^−2^, and fast reaction kinetics with a lower Tafel slope of 30 mV dec^−1^. Furthermore, Liu et al. designed a hierarchically structured NiCo_2_S@NiFe LDH supported on Ni foam as an efficient electrocatalyst for both OER and HER [41]. Benefiting from the engineered interface heterostructure and porous structure of conductive Ni foam, the as-obtained NiCo_2_S@NiFe LDH material demonstrated an outstanding OER catalytic activity with a lowest overpotential of 201 mV at a current density of 60 mA cm^−2^. DFT calculations also revealed that the ∆E_OH_ decreased as a result of the strong interaction and enhanced charge transfer between heterostructures, leading to an enhanced surface reactivity. With an open-cell 3D microporous structure, Ni foam can itself be transformed to an active OER electrocatalyst. Han et al. synthesized a 3D NiOx/NF through a facile in situ electrochemical oxidation process [134]. The as-obtained NiOx/NF has demonstrated a better activity than bare Ni foam with a measured overpotential of 390 mV at current density of 10 mA cm^−2^. The electro-oxidation process enabled the formation of active thin layers of NiOx on the surface of Ni foam. The enhanced electrochemical activity is attributed to the interface effect of NiOx/NF and the 3D nature of Ni foam.

Similar to Ni foam, carbon-based materials are also potential support materials for electrocatalysts. Carbon-based materials such as carbon cloth with demonstrated mechanical flexibility can be used to design efficient OER catalysts. Wang et al. developed a heterostructure containing hierarchical CoNi_2_S_4_@NiMn-LDH supported on carbon cloth as an efficient electrocatalyst for overall water splitting [135]. As an OER electrocatalyst, the material has demonstrated an overpotential of 269 mV at current density of 100 mA cm^−2^. The enhanced activity is mainly attributed to the material’s conductivity improvement, high stability, and superhydrophilic nature of carbon cloth support material. Carbon cloth with its fiber structure, higher conductivity, and higher surface area is highly preferred to construct catalysts for OER. He et al. constructed a 3D free-standing FeNi-LDH/CoP on carbon cloth for enhanced OER catalysis, where the supporting material improved the mass transfer and conductivity [136,137]. The as-obtained FeNi-LDH/CoP/CC has shown good catalytic activity with a lower Tafel slope of 33.5 mV dec^−1^, larger turnover frequency of 0.131 S^−1^, and higher current density of 350 mA cm^−2^ @ *η* of 254 mV.

### 3.4. Improving Stability of TM-LDHs

For commercial applications, the long-term stability of a given catalyst is top priority. One of the major challenges associated with the use of TM-LDHs for OER catalysis is their poor long-term stability [29]. To overcome this challenge, different techniques have been employed, including coating the surface of a catalyst with protective layers [138]. For instance, Obata and Takanabe reported a highly stable NiFeOx OER electrocatalyst with a protective layer working for over 96 h while maintaining its activity [138]. The surface of NiFeOx is covered by layers of CeOx that reduces the loss of Fe species from the main catalyst. They proposed that CeOx is permselective to the mobility of OH^−^ and O_2_, preventing the diffusion of redox ions and enhancing its stability. Furthermore, the stability of TM-LDHs can also be improved by growing TM-LDHs directly on 3D electrode materials, which reduce the loss of active sites [26]. Yang et al. designed a 3D hierarchical CoFe-LDH@NiFe-LDH supported on nickel foam, demonstrating outstanding stability as an OER catalyst under alkaline conditions [139]. Yin et al. also synthesized NiFe LDH directly on carbon materials through one-pot solution method [140]. Benefiting from the enhanced charge transport and the change in local electronic structure as a result of the conductive carbon support material, the catalyst has exhibited superior activity and stability. Furthermore, coupling layers of nanostructures could also enhance the activity and stability of TM-LDHs toward OER catalysis. Gao et al. synthesized CoFe LDHs decorated with CoO nanoclusters as an OER catalyst [141]. The strong electronic coupling between the layers could greatly enhance the stability of the interfacial structures. Despite all these measures, there is no specific method employed to obtain an electrocatalyst with outstanding activity and stability. There is a need to consider appropriate design strategies especially when planning to optimize activity and stability simultaneously.

## 4. First-Row TM-LDHs as OER Electrocatalysts

First-row TM-LDHs are potential alternatives to noble metal-based OER catalysts because of their abundance, lower cost, and comparable activities. Below, we summarize some of the recent developments on unary, binary, and ternary first-row-based TM-LDHs as an efficient OER catalyst. In addition, the overpotential applied to drive 10 mA cm^−2^ and Tafel slopes are compared (Table 3).

### 4.1. Unary TM-LDHs

Friebel et al. investigated γ-FeOOH as an OER catalyst and reported that the material has shown better activity compared to γ-NiOOH [142]. Other first-row TM-LDHs such as cobalt are also gaining attention for their catalytic applications. Cobalt hydroxide can exist in different forms and their OER activity differs significantly. Wang et al. reported that α-Co(OH)_2_ has a larger interlayer spacing compared to other forms of cobalt hydroxide such as β-Co(OH)_2_ and β-Co(OH)_2_ [143]. α-Co(OH)_2_ exhibited a lower overpotential of 60 mV compared to β-Co(OH)_2_ and β-CoOOH at a current density of 10 mA cm^−2^. Among the first-row transition metals, nickel and its derivatives have demonstrated promising results as an efficient electrocatalyst for water electrolysis in alkaline conditions [153]. Trotochaud et al. generated in situ electrochemically Ni-based LDHs with an outstanding OER performance [154]. The as-generated LDH demonstrated a lower overpotential of 336 mV at a current density of 10 mA cm^−2^ and a Tafel slope as low as 30 mV dec^−1^. Similar to Ni, Fe is also gaining attention as a potential catalyst for water electrolysis, owing to its abundance, low toxicity, and stability [36].

### 4.2. Binary TM-LDHs

Compared to monoatomic catalysts, multi-metal catalysts have shown higher catalytic activities because of synergistic interactions leading to a change in the electronic structure and adsorption energies of reaction intermediates [155]. Lu et al. reported that mesoporous NiFe nanosheets on nickel foam (NF) demonstrated a superior OER activity in contrast to monoatomic Ni or Fe on NF [156]. The material was synthesized through a facile electrodeposition method that required only 200 mV of overpotential to generate a current density of 10 mA cm^−2^. Compared to other TM-LDHs, NiFe-LDHs is the most researched OER catalyst because of its outstanding activity. Peng et al. employed a facile acid-etching method to engineer active sites on NiFe-LDH for enhanced OER catalysis [157]. The process induced the formation of edge Fe(OH)_3_ on NiFe-LDHs, proposed to react with Ni to form an active OER catalyst. The acid-etched NiFe-LDH demonstrated a lower overpotential, lower Tafel slope, and minimum charge transfer resistance compared to the untreated material. Yang et al. synthesized a binary LDH containing Co and Fe through coprecipitation method for OER catalysis [158]. A synergistic effect between Co and Fe was demonstrated in which Co_0.65_Fe_0.35_(OH)_2_ LDH showed an outstanding OER activity with a lower overpotential and good long-term stability. Among various first-row TM-LDHs, NiCo-based LDH is also potential option for OER catalysis with outstanding results. Li et al. designed a multilayer containing Ni_x_Co_y_ LDH hybridized with 3D NiCo_2_O_4_ with a core−shell nanowire array for OER catalysis [159]. The morphological feature enhances the surface area and conductivity of the material. The as-synthesized NiCo_2_O_4_@Ni_0.796_CoLDH/NF catalyst material has shown the lowest overpotential of 193 mV at a current density 10 mA cm^−2^. Furthermore, the material has also shown a lower Tafel slope of 37.59 mV dec^−1^ with remarkable OER kinetics in contrast to NiCo LDHs.

### 4.3. Ternary TM-LDHs

Previous reports have indicated that mixed-metallic compounds have shown coordinated enhancements among metallic ions toward OER catalysis [160]. Zhang et al. synthesized ultrathin nanosheets with thicknesses of 1.36 nm containing NiCoFe-LDHs for OER catalysis [150]. Compared to binary TM-LDHs such as NiFe-LDHs and NiCo-LDHs, the NiFeCo-LDH nanosheet demonstrated higher OER activities. The as-synthesized NiCoFe-LDH nanosheet showed the lowest overpotential of 288 mV to generate a current density of 10 mA cm^−2^ with a Tafel slope of 92 mV dec^−1^ as opposed to NiCo-LDH, which demonstrated an overpotential of 347 mV at a current density of 10 mA cm^−2^ with a higher Tafel slope of 108 mV dec^−1^. The incorporation of Fe into NiFeCo-LDH was reported to enhance the catalytic activity of the material for OER catalysis [161]. They fabricated a NiCoFe-LDH nanosheet with a unique nanostructure using the two-step hydrothermal method. The incorporation of Fe into NiFeCo-LDH resulted in the morphological change and oxidation of Ni, leading to the enhancement in the exposure of active sites. The optimized Ni_2_CoFe_0.5_-LDH/NF showed a lower overpotential of 240 mV to reach a current density of 10 mA cm^−2^, a lower Tafel slope of 65 mV dec^−1^, and an outstanding stability of 72 h for the continuous reaction under alkaline conditions. Recently, Lin et al. fabricated hierarchical NiFeCo-LDH supported on carbon fibers (CF) using a prominent metal−organic framework material called a zeolitic imidazolate framework [162]. The metal−organic framework was used as a source of metal Co and a precipitating agent for the metal ions. The as-obtained nanosheet had highly exposed active sites and an enhanced surface area. Cobalt doping into the composite improved the stability of the Fe local coordination environment and facilitated the π-symmetry bonding orbital in NiFeCo-LDH/CF. Thus, NiFeCo-LDH/CF exhibited an outstanding OER activity with a lower overpotential of 249 mV to generate a current density of 10 mA cm^−2^ and a stability of 20 h for continuous OER. The higher catalytic activity of NiFeCo-LDH/CF is attributed to the change in electronic structure in the composite as a result of Co doping inducing a stronger Ni3d-O2p and Co3d-O2p covalency. Furthermore, the conductive CF support material contributed to the enhanced flow of electrons across NiFeCo-LDH/CF.

## 5. Summary and Outlook

Electrocatalytic water splitting is a potential pathway to sustainable H_2_ production, a prominent gas with the highest energy density. However, the potential scalability is mainly hampered due to the extra overpotential required to drive the sluggish OER involving four-electron transfers. So far, different approaches have been put in place to obtain highly active and durable OER electrocatalysts based on TMs as an alternative to noble metals. TM-LDHs are at the center of interest as a potential candidate for water splitting catalysis owing to their morphological and electronic features, as well as their low cost and high abundance. However, TM-LDHs suffer from poor intrinsic activity and fewer active sites, limiting their applications in water electrolysis. Recently, many strategies have been designed and employed to improve the activities and stabilities of TM-LDHs toward OER catalysis. Herein, we provide a summary of current advancements made in the design of TM-LDHs to optimize their applications in OER catalysis. The basics of electrocatalytic water splitting have been presented, specifically focusing on the mechanisms of OER under alkaline conditions. We also summarized the fundamental parameters used to evaluate a given OER catalyst. Added to that, recently reported design strategies to optimize the activities of TM-LDHs such as structural and morphological engineering, composition tuning and electronic structure optimization, and coupling with conductive substrates are summarized. Despite rapid progress in the field and a high number of publications on TM-LDHs applications for OER catalysis, there still need to improve their activities to achieve the desired scalability. Therefore, the following critical issues must be considered to further improve the activity of TM-LDHs for OER catalysis:

(i) The activity of an OER catalyst is highly dependent on the exposure of active sites, which, in turn, is directly affected by the structural and morphological properties of the catalyst material. Thus, numerous strategies such as exfoliation, the creation of defects and pores, and altering the assembly of the surface structure have been designed and implemented. These strategies have their own advantages, and the choice of design strategy could have significance depending on the properties of the catalyst material. Although significant improvements have been recorded, challenges still exist regarding its practical applications, for instance, under harsh conditions. Therefore, designing an electrocatalyst rich with active sites and high stability is still a remaining challenge.
(ii) Composition tuning and electronic structure optimization are commonly employed as strategies to boost the catalytic activity of TM-LDHs. Doping is a potential alternative to change the composition and structure of TM-LDHs. This strategy can also be employed to enhance the surface electronic structure and coordination valence of TM-LDHs. Specially doping anions to TM-LDHs could lead to the enhanced adsorption of intermediates through altering the electronic structure of the adjacent active center. Therefore, combining TM-LDHs with both cation and anion dopants would be a potential strategy to design highly active and stable electrocatalysts for OER applications.
(iii) One of the innovative design strategies devised to overcome the poor conductivity of TM-LDHs is through coupling with conductive support materials. The most used conductive support materials for OER catalysis includes nickel foam and carbon materials. This strategy is effective in reducing the diffusion path length of ions during the electrochemical reaction, and it enhances the exposure of electrochemically active sites. Furthermore, the conductive support material can also act as an additional catalyst (nickel foam), can enhance the conductivity, and facilitates the ease of electron transfer. Therefore, the selection and application of appropriate conductive support materials during OER catalyst design could have potential contributions toward enhancing the OER activity and stability of a given catalyst.
(iv) Compared to unary TM-LDHs, multi-metallic catalysts have demonstrated higher catalytic activities because of the synergistic interactions leading to changes in the surface electronic structure, which will enhance the adsorption of reaction intermediates. However, this cannot always be true. Before designing a given OER electrocatalyst, investigating the synergistic effect between the metal atoms is paramount to obtain high-performing OER electrocatalysts.

## Figures and Tables

**Table 1:** Comparison of OER activities of exfoliated TM-LDHs (η is at 10 mA cm^−2^).

Catalyst	Exfoliation Method	Overpotential (mV vs. RHE)	Electrolyte	Ref.
NiCo LDH	O_2_-Plasma	367 mV	1 M KOH	[61]
FeNi LDHs	N_2_-Plasma	316 mV	1 M KOH	[73]
PA-ZnFeCo LDH	Liquid based	221 mV	1 M KOH	[74]
CoCo, NiCo and NiFe LDHs	Liquid based	300 mV	1 M KOH	[59]
NiCo-LDHs	Ar-Plasma	299 mV	1 M NaOH	[62]
N-CoFe LDHs	N_2_-Plasma	233 mV	1 M KOH	[71]
CoFe LDHs	Ar-Plasma	266 mV	1 M KOH	[69]
Ni-Co-F	O_2_-Plasma	300 mV	1 M KOH	[1]

**Table 2:** Comparison of OER activities of anion-doped TM-LDHs under alkaline conditions at current density of 10 mA cm^−2^.

Catalyst	Dopant	Overpotential (mV vs. RHE)	Electrolyte	Ref.
NiCo-LDH@HOS	S	293 mV	0.1 M KOH	[120]
NiFeS-2	S	286 mV	1 M KOH	[119]
Co_3_Fe LDHs-SF_6_	S	268 mV	1 M KOH	[122]
NiO@NiFe-LDH	N	265 mV	1 M NaOH	[123]
NiFe LDH	P	265 mV	1 M KOH	[115]
CoFe LDHs	S	233 mV	1 M KOH	[71]
CoFeP	P	305 mV	1 M KOH	[124]

**Table 3:** Comparison of OER activities of recent TM-LDHs in alkaline media at a current density of 10 mA cm^−2^.

Catalyst	Overpotential (mV vs. RHE)	Tafel Slope (mV dec^−^^1^)	Electrolyte	Ref.
γ-NiOOH	660	/	0.1 M KOH	[142]
γ-FeOOH	550	/	0.1 M KOH	[142]
α-Co(OH)_2_ LDH	400	130	0.1 M KOH	[143]
Co-LDH FNSAs	300	110	1 M KOH	[144]
NiFe LDH/ZiF-67	222	53	1 M KOH	[145]
NiFe LDH	320	/	1 M KOH	[146]
NiFe LDH/CNT	320	/	1 M KOH	[146]
Co_1.8_Ni LDH	290	66	1 M KOH	[147]
Co_0.8_Fe_0.2_OOH@C	254	33	1 M KOH	[148]
NiFeCo-LDH	297	33	1 M KOH	[149]
NiFeCo-LDH	288	92	1 M KOH	[150]
CoFeNi LDHs	195	53	0.1 M KOH	[151]
CoNiFe LDH	287	54.2	1 M KOH	[152]

## Data Availability

Not applicable.
